# Development and Validation of the Overall Foot Pain Questionnaire in Motorcycle Riders

**DOI:** 10.3390/ijerph17072233

**Published:** 2020-03-26

**Authors:** Israel Casado-Hernández, Ricardo Becerro-de-Bengoa-Vallejo, Marta Elena Losa-Iglesias, Alfredo Soriano-Medrano, Ángel Morales-Ponce, João Martiniano, Daniel López-López, César Calvo-Lobo

**Affiliations:** 1 Faculty of Health Sciences, Universidad Rey Juan Carlos, 28922 Alcorcón, Spain; israelcasado@yahoo.es (I.C.-H.); marta.losa@urjc.es (M.E.L.-I.); soriano.alfredo@gmail.com (A.S.-M.); clinicatorrijos@gmail.com (Á.M.-P.); 2 Facultad de Enfermería, Fisioterapia y Podología, Universidad Complutense de Madrid, 28040 Madrid, Spain; ribebeva@ucm.es (R.B.-d.-B.-V.); cescalvo@ucm.es (C.C.-L.); 3 Escola Superior de Saúde da Cruz Vermelha Portuguesa, 1300-125 Lisboa, Portugal; jmartiniano@esscvp.eu; 4 Research, Health and Podiatry Group, Department of Health Sciences, Faculty of Nursing and Podiatry, Universidade da Coruña, 15403 Ferrol, Spain

**Keywords:** foot diseases, reproducibility of results, sports, validation studies as topic

## Abstract

*Objectives*: Our primary aim was to develop a transcultural adaptation of a cycling questionnaire using the Borg CR-10 scale as a tool to describe the discomfort among motorcyclists during the riding process in two trial sessions. *Design*: A transcultural adaptation and descriptive cross-sectional study. *Settings*: Jarama motorcycling circuit (Madrid, Spain). *Participants*: The participants were riders recorded across in a final motorcycling race. Interventions: The study design is based in two tools, the adapted Motorcyclist Questionnaire (MQ-21) with 21 items and Borg CR10 Scale^®^ was used to determine discomfort level during motorcycling performance. The translation procedure, reliability, and reproducibility were performed. *Results*: All items showed an almost perfect intraclass correlation coefficient (ICC) (ICC = 0.909–1.00), except for item 9 (ICC = 0.881). Almost perfect internal consistency was shown for the total score (Cronbach α = 0.899). No systematic differences existed among test and retest in all items (*p* > 0.05) according to Bland–Altman plots. Respondents experienced slight discomfort on their body parts during the test-retest 1 h riding process. Foot discomfort was scored as 1.20, being the eighth of the 12 studied body parts. Conclusions: Internal consistency and test-retest reliability of the MQ-21 questionnaire were excellent and this questionnaire may be recommended to be used in motorcycling sports and clinical settings to evaluate the discomfort.

## 1. Introduction

According to the Federated Sports Statistics carried out by the Ministry of Education, Culture and Sports of Spain through the Higher Sports Council, motorcycling represented the 0.5% of the total federated sports licenses with 16,403 licensed pilots in 2016. Of these sports licenses, 95.8% corresponded to male federation licenses (15,719) and 4.2% to female (684). In Spain, the Autonomous Communities that presented the highest percentage of federated pilots were Catalonia with 17.2%, followed by Andalusia with 14.3%, and by the Community of Madrid with 12.6% of the total.

In Spain, the motorcycling boom grew up to 17% more in 2016 according to the Asociación Nacional de Empresas del sector de Dos Ruedas (ANESDOR). Regarding the marketing, motorcycles accounted for 88% of the total marketing of two wheeled vehicles in 2016, including 154,075 registered units, 17.1% more than the previous year, scoring its best result since 2008. By displacement segments, motorcycles of less than 125 cc accounted for 59% of the marketing, with 91,449 registered units, and an increase of 14.2%. Nevertheless, the high displacement bikes registered the highest growth (+21.6%) and comprised 62,625 registered units in 2016 in Spain [1].

During 2016, a total of 160,978 motorcycles were registered, including 23,755 motorcycles of more than 750 cc. During the same year, a total of 715,368 new licenses for motorcycle circulation were recorded. The total number of motorcycles registered in Spain until 2016 was 3,211,474 [2].

These data highlighted the number of motorcycle users that may directly influence climate change and global warming related to congestion on roads and cities [3].

Motorcycling injury research may be broadly divided into two categories, trauma-related (as a result of collision or fall) and non-trauma-related (over-use type injuries). Motorcycling injury literature is primarily focused on lower extremities (44.3%) and upper extremities (18.6%) with a lack of published data on foot injuries. Khanna et al. reported lower limb injuries accounted for 43% of the total extremity injuries. Most of the ligamentous injuries were strains of the collateral ligaments of the knee. Ankle and foot were the most commonly involved areas in fractures and dislocations (36%), half of these fractures being located at phalanges and metatarsals [4].

There are studies that suggest that there is a relationship in motorcycle commutes with the cost in travel and energy consumption before and after riding on a motorcycle [5]. Another study related the incidence of risk of injuries in motoriders involved in road crashes to the roads [6].

On the other hand, there are several studies about the relationship between foot pain and cycling [7,8]. For their research, a survey link was distributed focusing on foot injuries.

Nevertheless, there is a lack of research regarding the frequency, etiology, and/or management of foot pain in motorcycling, which may be a great difficulty for clinicians. Indeed, this poorly available literature comprised descriptive nonsystematic literature reviews or point of view articles located in no peer-reviewed journals. Therefore, we carried out a study to address the lack of information about discomfort in motorcycling particularly focused at the feet. Firstly, our primary aim was to develop a transcultural adaptation of a cycling questionnaire using the Borg CR-10 scale as a tool to describe the discomfort among motorcyclists during the riding process in two trial sessions. Secondly, the goal of this study was to assess foot pain or discomfort of feet while riding a motorcycle.

## 2. Material and Methods

### 2.1. Ethical Approval

The investigation was favorably reported by Ethical Committee of University Juan Carlos of Madrid (Spain) with internal register number: 050520165316. All participants agreed to conduct the study and informed consent was signed. Furthermore, our investigation tools complied with the standard ethical rules and human investigation guidelines of the World Medical Association approved by the Declaration of Helsinki.

### 2.2. Study Design

The Borg CR-10 scale and a transcultural adaptation and descriptive cross-sectional investigation was performed out in order to develop a cycling questionnaire using the Borg CR-10 scale as a tool to describe the discomfort among motorcyclists during the riding process, as well as assess foot pain or discomfort of feet while riding a motorcycle.

### 2.3. Participants

Participants in this study were riders across Spain who presented for a final motorcycling race competition at in the Jarama motorcycling circuit (Madrid, Spain). The study was performed in July 2018.

The criteria for inclusion were aged between 18 and 55 years, being an active rider at the time of the survey with at least one performance weekly, and with a minimum activity of 1 h/wk of motorcycling for at least the past 12 months and who have driven in speed circuit touching with the knee in the curve step. Exclusion criteria were participants who had showed previous experience related with surgery trauma records in the past 2 years, neurologic alterations, as well as those who rejected to sign the consent document or were unable to understan the rules necessary to carry out the actual investigation.

### 2.4. Outcome Measurements and Procedure

**Figure 1:**
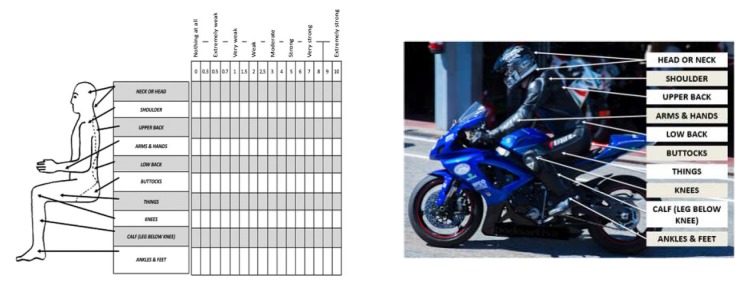
The left side of the body chart discomfort using the Borg CR-10 scale and the right side of the moto rider using the Borg CR-10 scale.

The Borg CR-10 tool, which may be considered as a measuring instrument for malaise, was used to analyze each participant’s level of malaise during physical work and the perception of prolonged exertion. The Borg CR-10 scale and a body chart (Figure 1) were presented in order to permit subjects to indicate which parts of their body experienced discomfort i.e., (1) head/neck, (2) shoulder, (3) back, (4) arm, (5) lumbar, (6) buttocks, (7) back thigh, (8) knee, (9) calf, (10) ankle, (11) foot and how much malaise was felt, on a scale from 0 to 10; 0 being no discomfort and 10 being extreme discomfort [9]. A lower Borg CR10 Scale^®^ score represented a higher level of function or no discomfort.

The Borg CR10 Scale^®^ used was identical in our research [10] due to the type of the questionnaire and analogous psychometric properties like the Visual Analog rating Scale (VAS) [11,12].

Furthermore, this scale has been used in another study as a measuring tool for discomfort in leg [10] and during motorcycling practice and while motorcycling [13,14].

Previously, we adapted the used test to assess comfort on motorcycles [13] of motorcyclists with 10 items but the foot was not included. We added item 11 asking for foot comfort and item 12 to find out if discomfort of the foot reduces the motorcyclist’s performance.

Between consecutive participants the test-retest reliability was performed referring to a single sports club of motorcyclists at Madrid (Spain). In our study a heterogeneous sample was chosen since the purpose of the measurement was to be used in multiple conditions.

The adapted Motorcyclist Questionnaire (MQ-21) with 21 items and Borg CR10 Scale^®^ were recorded twice. All participants underwent two testing sessions separated by at least 15 days apart. All measurements were recorded by the same researcher. After completion of the 1-h motorcycling period, the subjects were asked to give ratings about discomfort while motorcycling using the Borg CR-10 scale each session.

### 2.5. Translation Method

The translation method was performed according to the guidelines suggested by the group research of the Beaton et al. [15,16] following principles of good criteria manners that was recorded by the ISPOR [17].

In summary, the steps carried out were

(a) Request to the author, Borg. G. of the original questionnaire to translate the original questionnaire Borg CR10 Scale^®^ and Scales with instructions, which may be obtained from borgperception@telia.com, and the foot and cycling questionnaire permission was given by Hayley Uden from University of South Australia, Australia [18] that we adapted to MQ-21.
(b) New translation: two freelance bilingual physicians from Spain (native Spanish speakers) translated the MQ-21 and Borg CR10 Scale^®^ into Spanish. In addition, the main author adapted the questionnaire and was part of the translation at all times according to the suggestions above [17].
(c) Accommodation: a meeting was undertaken to find any discrepancies in the new translations with every translator separately. A written memory documented the translation procedure.
(d) The accommodate new translated version of the MQ-21 and Borg CR10 Scale^®^ was translated again to English by other two native English speaker physicians. The translators were blinded and did not seen the version of the MQ-21 and Borg CR10 Scale^®^.
(e) The new translated version and the original version were compared to guarantee the analogy of the version translation, and any difference or unsure phrasings were adapted.
(f) Harmonization: the unify group was composed by the preceding and onwards translators, a investigation physician, and a performer language expert.

The members met with the main author separately. Then, the new version of the MQ-21 and Borg CR10 Scale^®^ was submitted through a collective e-mail postbag.

All the collective partners agreed to the new translated version.

(g) Cognitive meetings of the new version were realized in a privy physician praxis. Eight physicians and eight non-clinicians, with no foot disorders, took part. The main author, retrospectively, accomplished the cognitive polls orally. Firstly, each participant accomplished the MQ-21 and Borg CR10 Scale^®^ and was afterwards questioned about the purpose of the item and their reply to supply potential mistakes and problems to reduce future mistakes and non-reply [19].
(h) The outcomes from the deliberate interviews were analyzed and a closed adapted version was approved. Any arguments as to the composition of the MQ-21 and Borg CR10 Scale^®^ were provided in writing [17].
(i) The concluded version was edited, probed for spelling and grammar arguments, and disposal was finished.
(j) An end report kept a record of the translation method. A language domain of Spanish secondary school scale was the purpose of the latest translation.

### 2.6. Sample Magnitude

Once test-retest reliability was analyzed, for enforcement in particular subjects and for utilization in clinical praxis, a high-rise value of intraclass correlation coefficient (ICC), 0.9–0.95, was accepted owing to the probability of increased measurement reliability [20,21].

A 0.90 ICC result and a ± 0.1 confidence interval was considered, for a sample size of 35 subjects, enough to realize statistical measurements [22].

The study size was consciously selected to be heterogeneous as the assessment device was designed to be used for various settings.

Eleven items were selected for our research due to their relationship in foot disorders and Borg CR10 Scale^®^. The other nine items were not relevant in the study. These items were used as control for future research.

### 2.7. Statistical Analysis

All data were explored for normality using the Kolmogorov–Smirnov test, and data were considered normally distributed if *p* > 0.05. Descriptive statistical analysis was performed using mean ± SD and a 95% confidence Interval (95% CI).

The intra-class correlation coefficient (ICC) by a two-way random effects model (2.1), single measures, and absolute agreement, was used to express reliability and evaluate the test-retest reliability of each gait parameter. To interpret ICC values, we used benchmarks as proposed by Landis and Koch [23]: 0.20 or less, slight agreement; 0.21 to 0.40, fair; 0.41 to 0.60, moderate; 0.61 to 0.80, substantial; and 0.81 or greater, almost perfect [23].

The internal consistency was determined by calculating the Cronbach’s alpha, with 0 indicating no internal consistency and 1 corresponding to perfect internal consistency.

To compare groups an independent *t*-test or Kruskal–Wallis test was used according to normal or no normal distribution of data. Additionally, paired *t*-tests or a Wilcoxon signed-rank test were performed to test for any systematic differences between sessions, if the data had a normal or non-normal distribution, respectively.

In addition, Bland–Altman designs were employed to check compliance and heteroscedasticity [23].

A *p* value < 0.05 with a CI of 95% was considered statistically significant for all tests (SPSS for Windows, version 20.0; SPSS Inc., Chicago, IL, USA).

## 3. Results

### 3.1. Descriptive Data

There were 60 participants and 12 of them did not show up at retest or the second session. Finally, test-retest reliability was performed among 48 participants, all of them practicing the sport of motorcycling in a motorcycle sport club in Madrid (Spain).

Sociodemographic data of the total group showed an age mean of 37.66 ± 6.11 years with a 95% CI ranging from 35.93 to 39.39 years, a weight mean of 73.58 ± 7.11 kg with a 95% CI ranging from 71.57 to 75.59 kg, a height mean of 175.54 ± 5.30 cm with a 95% CI ranging from 174.04 to 177.04 cm, and a body mass index (BMI) of 23.83 ± 1.50 kg/cm^2^ with a 95% CI ranging from 23.40 to 24.26 kg/cm^2^ (Table 1).

All participants underwent two testing sessions separated by 16.96 ± 1.32 days (range, 15–20). The sociodemographic subject characteristics are shown in Table 1. The educational level of participants was as follows: 15 participants who finished high school, 14 participants who completed a college degree, and 19 participants who completed a university degree. Participants completed a paper version of the MQ-21 using Borg CR10 Scale^®^. Twelve dropouts were documented. The time for replying the MQ-21 using the Borg CR10 Scale^®^ varied between 8 and 11 min at the first session.

No issues were detected in the ahead translations, and in the process there was good compliance between the two periods. Only low disagreement was detected, consisting of contrasting synonyms and contrasting values of prepositions. The rear translations were employed as control and were the same in most of the items, and nearly in the initial English version.

The physical interviews reported no difficulties in participant’s understanding and apprehension of the Borg CR10 Scale^®^ and therefore no adjustments were made to the terminology and the icons in the survey.

All the variables showed a non-normal distribution (*p* < 0.05) except age, weight, height, and BMI that showed a normal distribution (*p* > 0.05).

The type of motorcycle and the reason why the motorcycle is used in Spain are described in Table 2.

### 3.2. Reliability and Reproducibility

The reliability data for the test and retest are presented in Table 3 and all items showed an ICC almost perfect, higher than 0.90, ranging from 0.909 to 1.00, except for the item 18 that had the lowest value with 0.881. Almost perfect internal consistency was shown for the total score with Cronbach Alpha of 0.899. No systematic differences existed among test and retest in all items (*p* > 0.05) that are shown in Table 3.

The results of total mean of Borg’s scale discomfort ratings by the respondents at the test and retest sessions are presented in Table 3. The results represent the rating of discomfort level on each of the body parts at head-neck, shoulder, back, arm, lumbar, buttocks, back thigh, knee, calf, ankle, and foot during 1 h in each test.

The results show that the respondents experience slight discomfort on their body parts during test-retest 1 h riding process. Head/neck and back showed the highest discomfort with a rating of 2.7 and 2.18, respectively, and both ankle and calf showed the lowest discomfort with rating of 0.85. Foot discomfort was 1.20 being eighth of the 12 studied body parts.

**Figure 2:**
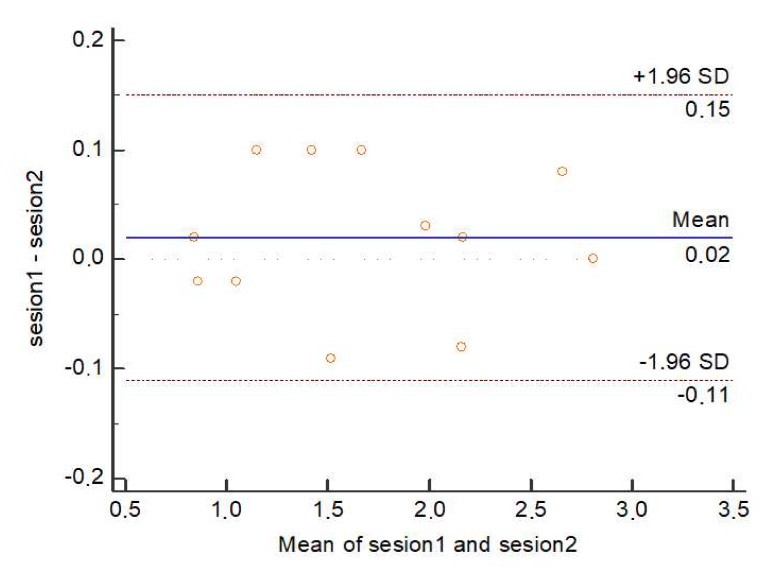
Bland–Altman plot comparing test-retest results for individual participants.

There was no significant or suitable variation in Bland–Altman charts displayed from test to retest in total score with mean differences within the 95% confidence interval of all measurements below 1 point (Figure 2).

## 4. Discussion

The purpose of this study was to achieve a transcultural adaptation of a cycling questionnaire using the Borg CR-10 scale as a tool to describe the discomfort among motorcyclists during the riding process.

This paper is the first known study to describe and report foot discomfort in motorcyclists in Spain and presents a survey on the perception regarding riding discomfort among motorcycle riders in Spain and its reliability for use in sports and clinical settings.

The questionnaire rates the experienced discomfort in motorcyclist at different parts of the body but mainly at the foot, as there is no literature about the discomfort of the foot while riding in this population.

Prolonged motorcycle riding has previously been identified as a potential source of motorcyclist discomfort due to reasons of improper structural motorcycle design, imbalance of engine inertial, and also due to road excitation and the vibration source on the foot pedal which has been found to be the main factor that contributes to the highest discomfort level among motorcycle teenagers riders in Malaysia [24].

There is a lack of knowledge regarding these issues and we did not find peer-reviewed research dealing with discomfort among motorcyclists, including the foot.

In our study the motorcyclist experienced discomfort of the foot to be the eighth position of discomfort after head/neck, back, lumbar, arm, knee, shoulder, and buttocks. Foot discomfort while riding was worse than the back thigh, calf, and ankle.

Our study highlights the value of considering the foot on the footpeg as a mechanism of generating pain. In this survey, we found that participants reported a low foot discomfort score of 1.20 ± 1.91, but, surprisingly, the participants answered at item 12 that the foot discomfort level bothers them but they can still drive the motorcycle.

Uden et al. concluded in their research a high frequency of foot pain in cycling with special incidence in the forefoot being the main reason at the foot–pedal interface. They managed to decrease foot pain by changing shoes, checking the fit of shoes, and moving the toes in the shoes [18].

Priego Quesada et al. related, in their research, that the knee was the body region most injured in cycling. In their study they included the foot as a body region injury but there were no results about it [8].

Casado et al. analyzed the plantar pressure in motorcycling with foot insoles of different hardness. All the participants mentioned higher comfort with hard insoles and the lowest values in plantar pressure. Their conclusion was that hard insoles reduce plantar pressures due to the impact and vibration in the foot on the footpegs, increasing comfort and performance [25]. Metatarsal fractured bones was the most common foot injury in motorcycle trauma [26].

The main age of participants in our study were under 40 (37.66 ± 6.11) and sport motorcycle was the most used. The conclusion of De Rome et al. in their research was that riders between 20 and 25 years old had the highest risk in crash and injury trauma due to the frequency of commuting by bike and hours ridden per week. In their research the sport motorcycle was the most used [27].

Studies reported that boots motorcycling clothes reduce the risk of suffering lower limb injuries [28] and more than 50% of fractures due to the knee-high and ankle form of the boot [29]. Motorcycle boots are used to protect knees.

A global approved translation form was used to contribute a Spanish adaptation of the questionnaire for motorcyclists and translation was achieved using meticulous methodology to ensure the survey was transcribed and accommodated to Spanish motorcyclist situations [16].

There is no consensus about the preferred sample range for analytical interviewing [15,17]. In this study, the analytical interviews were designed as a one-on-one verification in order to probe if defendants understood the survey and replied to the translated survey in agreement with the meaning to adapt the test used previously to assess foot pain and cycling [18]. Due to the relatively simple survey, a high-scale physical interview was not identified as needed [17] and a sample range of 48 was assumed adequate.

### 4.1. Reliability and Reproducibility

The reliability and reproducibility of the MQ-21 was established to be excellent. Additionally, the Bland–Altman charts did not show contrasts among test and retest, showing good agreement.

### 4.2. Limitations

Firstly, a limitation of the study is the cross-sectional research design which does not allow for cause and effect relationships to be established. Although this paper concentrates specifically on the foot discomfort, many other factors not explored within this research could be attributed to the presence of foot discomfort whilst riding. These could include motorcycle set-up as saddle height, saddle distance or cleat position; type of motorcycling boot as material of sole; shoe or boot fit; foot type as pes planus or pes cavus; presence of any lower limb biomechanical or structural deformities. None of these factors can be confirmed or refuted with the data from this research. Nevertheless, this data provide support for these factors to be explored.

Due to the sample size of the survey, external generality of our findings may not be assumed; and a large survey should be carried out.

Within these populations, given the frequency of foot discomfort reported, investigations into causation and management of foot discomfort is warranted, which may involve an investigation of motorcycling shoe wear and/or the foot–pedal interface or foot type. More effort also should be directed on improving the foot discomfort in order to provide comfort at the optimum level.

Moreover, a large sample and case control studies are needed using this novel questionnaire to detect minimal clinical differences.

## 5. Conclusions

The internal consistency and test-retest reliability of the MQ-21 questionnaire were excellent and this questionnaire may be recommended to be used in motorcycling sports and clinical settings to evaluate the discomfort. Related to the foot, this research found a prevalent foot discomfort in motorcyclists that bothers them, but they can still drive the motorcycle.

## Figures and Tables

**Table 1:** Socio-demographic characteristics of the sample population.

Individal Demographics	Total Group Mean ± SD Range *N* = 48	Men Mean ± SD Range *N* = 45	Women Mean ± SD Range *N* = 3	*p* Value
Age (years)	37.66 ± 6.11 (35.93–39.39)	37.68 ± 6.12 (35.89–39.47)	37.33 ± 7.37 (19.02–55.64)	0.942 *
Weight (kg)	73.58 ± 7.11 (71.57–75.59)	74.62 ± 6.00 (72.86–76.37)	58 ± 2.64 (51.44–64.56)	0.001 *
Height (cm)	175.54 ± 5.30 (174.04–177.04)	176.31 ± 4.51( 174.99–177.62)	164 ± 1.00 (161.52–166.48)	0.001 **
BMI (kg/m^2^)	23.83 ± 1.50 (23.40–24.26	23.98 ± 1.41 (23.56–24.39)	21.56 ± 0.96 (19.18–23.94)	0.001 *

Abbreviations: kg, kilograms; cm, centimeters; BMI, body mass index; SD, standard deviation. In all the analyses, *p* < 0.01 (with a 95% confidence interval) was considered statistically significant. * *p* value from independent *t*-test. ** *p* value from Kruskal–Wallis test.

**Table 2:** Type and reason for motorcycling.

Type of Motorcycle	*n* (%)	Reason for Motorcycling	*n* (%)
Sport	35 (72.91%)	Work	4 (8.33%)
Turismo	4 (8.33%)	Competition	8 (16.66%)
Naked/Seminaked	5 (10.41%)	Recreation	35 (72.91%)
Trail	1 (2.08%)	Other	1 (2.08%)
Custom	1(2.08%)		

**Table 3:** Results of reliability, test-retest, and systematic differences of the Spanish of performance and discomfort in the motorcycle driving questionnaire according to each question.

Comfort Items	Test (*n* = 48) Mean ± SD (95% CI)	Retest (*n* = 48) MEAN ± SD (95% CI)	Total Mean ± SD (95% CI)	ICC (95% CI)	*p* Value
**Item 1:** Indicates according to a scale of discomfort, 0 being no pain and 10 very painful, what part has bothered you ever driving the motorcycle (head/neck)	2.70 ± 2.96 (1.87–3.54)	2.62 ± 2.87 (1.81–3.43)	2.66 ± 0.05 (2.62–2.69)	0.991 (0.985–0.995)	0.874
**Item 2:** Indicates according to a scale of discomfort, 0 being no pain and 10 very painful, what part has bothered you ever driving the motorcycle (shoulder)	1.47 ± 2.52 (0.76–2.19)	1.56 ± 2.63 (0.81–2.30)	1.51 ± 0.06 (1.47–1.55)	0.995 (0.992–0.997)	0.970
**Item 3:** Indicates according to a scale of discomfort, 0 being no pain and 10 very painful, what part has bothered you ever driving the motorcycle (back)	2.18 ± 2.80 (1.39–2.98)	2.16 ± 2.77 (1.38–2.95)	2.17 ± 0.05 (2.16–2.17)	0.987 (0.977–0.993)	0.968
**Item 4:** Indicates according to a scale of discomfort, 0 being no pain and 10 very painful, what part has bothered you at any time driving the motorcycle (arm)	2.00 ± 2.55 (1.27–2.72)	1.97 ± 2.52 (1.26–2.69)	1.98 ± 0.02 (1.97–1.99)	0.982 (0.969–0.990)	0.874
**Item 5:** Indicates according to a scale of discomfort, 0 being no pain and 10 very painful, what part has bothered you ever driving the motorcycle (lumbar)	2.12 ± 2.52 (1.41–2.83)	2.20 ± 2.62 (1.46–2.95)	2.16 ± 0.05 (2.12–2.19)	0.971 (0.947–0.983)	0.799
**Item 6:** Indicates according to a scale of discomfort, 0 being no pain and 10 very painful, what part has bothered you ever driving the motorcycle (buttocks)	1.47 ± 2.07 (0.89–2.06)	1.37 ± 1.94 (0.82–1.92)	1.42 ± 0.07 (1.38–1.46)	0.947 (0.905–0.970)	0.956
**Item 7:** Indicates according to a scale of discomfort, 0 being no pain and 10 very painful, what part has bothered you ever driving the motorcycle (back thigh)	1.04 ± 1.94 (0.49–1.59)	1.06 ± 1.76 (0.56–1.56)	1.05 ± 0.01 (1.04–1.05)	0.963 (0.935–0.979)	0.839
**Item 8:** Indicates according to a scale of discomfort, 0 being no pain and 10 very painful, what part has bothered you ever driving the motorcycle (knee)	1.72 ± 2.57 (1.00–2.45)	1.62 ± 2.43 (0.93–2.31)	1.67 ± 0.07 (1.63–1.72)	0.988 (0.978–0.993)	0.958
**Item 9:** Indicates according to a scale of discomfort, 0 being no pain and 10 very painful, what part has bothered you ever driving the motorcycle (calf)	0.85 ± 1.99 (0.28–1.41)	0.87 ± 1.86 (0.34–1.40)	0.86 ± 0.01 (0.85–0.86)	0.881 (0.787–0.933)	0.956
**Item 10:** Indicates according to a scale of discomfort, 0 being no pain and 10 very painful, what part has bothered you ever driving the motorcycle (ankle)	0.85 ± 1.89 (0.31–1.38)	0.83 ± 1.86 (0.30–1.35)	0.84 ± 0.01 (0.83–0.84)	0.909 (0.838–0.949)	0.780
**Item 11:** Indicates according to a scale of discomfort, 0 being no pain and 10 very painful, what part has bothered you at any time driving the motorcycle (foot)	1.20 ± 1.91 (0.66–1.74)	1.10 ± 1.74 (0.61–1.59)	1.15 ± 0.07 (1.11–1.19)	0.924 (0.864–0.957)	0.461
**Item 12:** About the foot. This discomfort or pain (select + from 1 reply). Does not let me ride a motorcycle = 1; reduces my performance = 2; it bothers me but I can still drive the motorcycle = 3; it does not bother me at all = 4; other = 5	2.81 ± 0.73 (2.60–3.02)	2.81 ± 0.73 (2.60–3.02)	2.81 ± 0.73 (2.60–3.02)	1.000 (1.000–1.000)	1.000

Abbreviations: SD, standard deviation; CI 95%, confidence interval 95%; ICC, intraclass correlation coefficient. *p* values from Wilcoxon signed-rank test.
